# Enhancing prediction of short linear protein motifs with Wregex 3.0

**DOI:** 10.1016/j.csbj.2024.07.013

**Published:** 2024-07-17

**Authors:** Gorka Prieto, Jose A. Rodríguez, Asier Fullaondo

**Affiliations:** a Department of Communications Engineering, University of the Basque Country (UPV/EHU), Bilbao, Spain; b Department of Genetics, Physical Anthropology and Animal Physiology, University of the Basque Country (UPV/EHU), Leioa, Spain

**Keywords:** Protein motif finding, Post-translational modification, Decoy database

## Abstract

Short linear motifs (SLiMs) play an important role in protein-protein interactions. However, SLiM patterns are intrinsically permissive and result into many matches that occur just by chance, specially when targeting large datasets. To prioritize these matches as candidates for functional testing, we developed Wregex (Weighted regular expression), which uses a position-specific scoring matrix (PSSM) to order a list of regular expression matches according to a PSSM-derived score. Here we present Wregex 3.0, an improved version with new functionalities such as the support for a second auxiliary motif to help refining prediction of a primary SLiM, and post-translational modifications (PTMs) enrichment taking into account that many regulatory SLiM-mediated interactions are modulated by one or more PTMs. This version also incorporates a number of new features such as a convenient use of subproteomes, showing UniProt annotations such as disordered regions, searching for all known motifs and generating decoy databases for enrichment analysis. We provide case studies to illustrate how these new Wregex functionalities enhance prediction of short linear protein motifs. The Wregex 3.0 server is freely accessible at https://ehubio.ehu.eus/wregex3/.

## Graphical abstract

**Figure:**
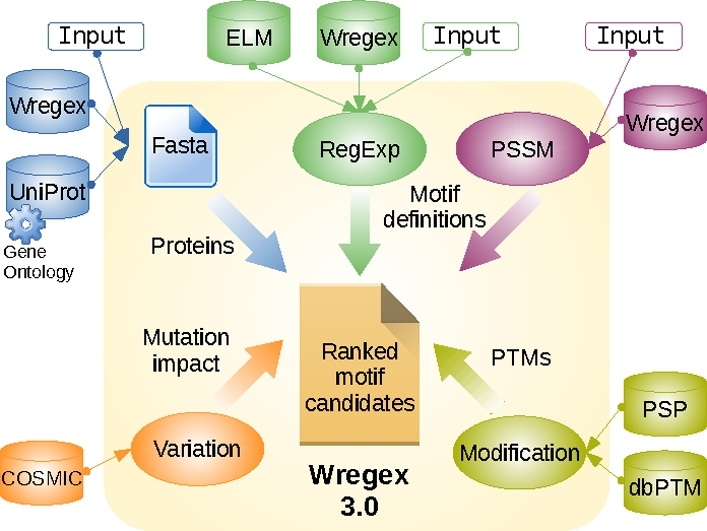

## 1 Introduction

Short linear motifs (SLiMs) are short stretches (commonly 3 – 15 residues long) of protein sequence that participate in protein-protein interactions (PPIs). Not requiring a stable tertiary structure to be functional, these motifs are typically enriched in intrinsically disordered regions (IDRs) [1]. SLiMs usually contain a few conserved amino acids that interact with the ligand domain, but different spacing between these conserved locations, as well as the presence of different residues with similar physicochemical properties are often allowed. SLiMs are commonly defined and searched for using regular expressions, an approach used in programming to define text patterns. In this regard, the motif patterns for hundreds of SLiMs can be found in the Eukaryotic Linear Motif (ELM) database [2], which is a highly valuable resource in SLiM research being both a repository of annotated motif data as well as an exploratory tool for motif prediction.

However, SLiM patterns are intrinsically permissive. As a result, searching for these motifs, in particular when targeting large datasets, such as a proteome, results into many matches that occur just by chance, and are functionally irrelevant. This issue is well-documented [3], so it is crucial to prioritize matches that are more likely to be functional. With the objective of prioritizing these matches for subsequent experimental testing, we created Wregex (Weighted regular expression) [4], a web tool that combines a regular expression with a position-specific scoring matrix (PSSM), to provide a ranked list of motif matches according to a PSSM-derived score. Wregex provides predefined PSSMs for some motifs and also allows the user to upload a training dataset to easily build a custom PSSM for any other motif. Other web-based tools that complement pattern matches with a range of attributes such as interactions data, predictions of disordered regions or intra-domain biological features are ScanProsite [5], SLiMSearch [6] and SLiMAn [7]. Each of these tools has a specific purpose and complement each other. A comparison between these tools is available in Supplementary File S1.

Considering that mutations that cause cancer and other human diseases may affect SLiM function, we subsequently developed Wregex 2.0 [8], which allows predicting the impact of cancer-related missense mutations in candidate motifs, and ranking SLiMs according to the impact and prevalence of each mutation. Furthermore, the speed performance of Wregex methodology allowed us to analyze the whole human proteome for the impact of every mutation reported in COSMIC (Catalogue of Somatic Mutations in Cancer) [9] in the matches of every motif defined by ELM [2].

**Fig. 1:**
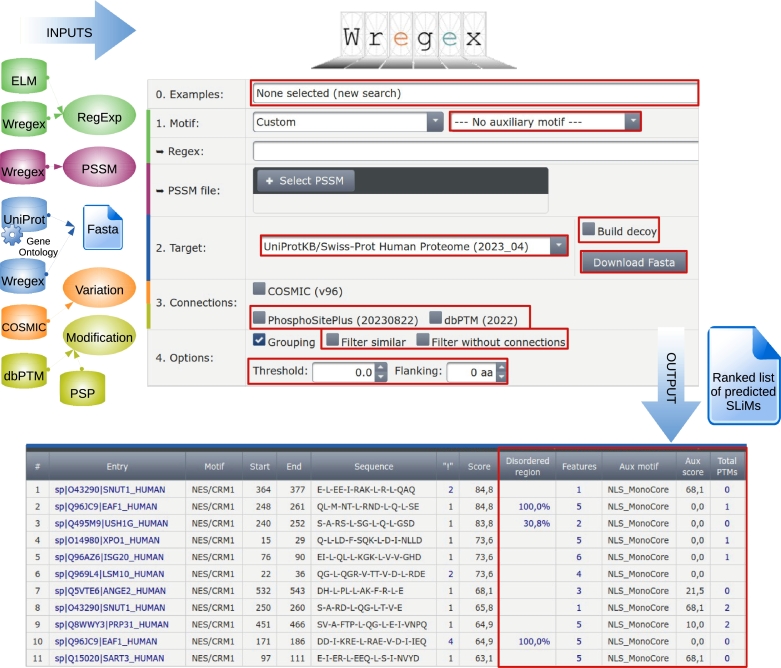
Wregex 3.0 at a glance. Key technologies and external database connections are depicted over the motif search form. Improvements and new functionalities of this new version are highlighted in red.

Here we present Wregex 3.0, a significantly improved version with updated databases and new functionalities (see Fig. 1). Some of these new functionalities are directed to improve user experience. Regarding protein input, now target proteins can be specified in formats other than fasta. For example a list of protein accessions or gene names can be entered manually or uploaded from a local file, and the corresponding fasta sequences are obtained automatically and also made available for downloading. It is also possible to enter a gene ontology (GO) term [10], [11], and a list of revised human proteins is retrieved dynamically from UniProt [12]. In addition, it is now possible to specify a number of flanking amino acids to be displayed at both sides of the motif match. We have used this feature to analyze the charge distribution of the four amino acid residues C-terminal to putative NESs (nuclear export signals) predicted in proteins differentially exported in cells expressing a cancer-related mutant form of the nuclear export receptor CRM1/XPO1 [13]. Another new option allows the user to indicate whether identical sequence matches in different positions or proteins should be filtered. This can be useful when further assays will be carried out using only the sequence matched by the motif instead of the full protein sequence. Additionally, an *Examples* dropdown widget has also been included as an starting point to load sample data corresponding to the different functionalities presented here and get familiar with the results.

More importantly, we have included several functionalities that enhance prediction relevance. For example, there are biological contexts where the activity of a motif is impacted by the presence of a second, functionally-related, motif in the same protein. We reasoned that the simultaneous search for both motifs (which we refer to as “primary” and “auxiliary” in terms of search) could therefore improve prediction of functionally relevant motifs. We illustrate this new functionality with a case study using two SLiMs related to nucleocytoplasmic transport: the nuclear export signal (NES) as the primary motif and the nuclear localization signal (NLS) as the auxiliary motif. We corroborate that the presence of a NLS match is useful to predict better NES candidates.

On the other hand, considering that many SLiM-mediated interactions are modulated by one or more post-translational modifications (PTMs), we have included support for enriching candidate reports with PTM information from PhosphoSitePlus (PSP) [14] or dbPTM [15]. To illustrate this new Wregex feature we include a case study where PTM information is used to build a custom PSSM to improve the prediction performance. In addition, we have created an interactive chart (available at the *Charts* tab) with the results of analyzing the presence of every PTM in every ELM motif match in the whole human proteome.

Furthermore, considering that SLIMs are typically enriched in intrinsically disordered protein regions (IDRs) we now report whether the positions of the matched sequences fall within a disordered region. This information is obtained from the UniProt [12] human proteome, and displayed as the percentage of the matched sequence overlapping with an IDR. Additionally, we provide an overview of other features annotated in UniProt that intersect with the positions of the matched sequence. These features include, among others, regions of interest, compositionally biased regions, binding sites, PTMS, secondary structures, domains and variants.

To test how the new Wregex functionalities can enhance prediction, we have used a decoy-based approach, inspired by the target-decoy approach [16] commonly used in shotgun proteomics to distinguish correct from incorrect identifications. Wregex 3.0 offers the possibility to easily generate a decoy version of any target protein dataset by selecting the new *Build decoy* option. This will automatically build a fasta file by shuffling the amino acid sequences of the selected target dataset. Our results show that target/decoy comparisons represent a useful approach to measure the enrichment effect of different strategies in SLiM searching.

## 2 Experimental section

### 2.1 Selection of target proteins

Wregex can be used with any target proteins by either selecting a fasta file stored in the user's computer or by pasting the fasta sequence into the target input box of the search form. Besides, we provide a convenient access for the case of the human proteome by selecting the corresponding entry in the *Target* dropdown menu. The user can select the entire human proteome from UniProt, a list of UniProt protein accessions or a list of official gene symbols.

When the entire human proteome is selected, it is searched using a local copy of the UniProt human proteome stored in our server for performance reasons. This local copy is also used when selecting a list of protein accessions or gene names. When a given protein accession or gene name entered by the user is not found in our proteome copy, a message is shown to the user. The version of our proteome copy is always displayed to the user, so a manual fasta entry can be selected in case of needing a different proteome version. The UniProt version used for the different case studies in this paper is 2023_04.

Wregex also facilitates targeting a human subproteome by entering a GO term, in which case a dynamic query is sent directly to UniProt. This search is equivalent to using UniProt advanced search and selecting the provided GO term as Gene Ontology [GO], Homo sapiens (Human/Man) [9606] as Organism [OS], and Yes as Reviewed.

### 2.2 Decoy database generation

We have included in Wregex 3.0 the option to build a decoy database from any given target database. This allows to test if the number of predicted SLIMs is significantly larger in the target database than in the decoy one, thus providing information on the discriminative power of a SLiM. We build decoy sequences by shuffling the amino acids of target sequences, thus preserving the proportion of each amino acid and the lengths of the proteins. This shuffling is carried out starting at the second amino acid in order to preserve the prevalence of methionine as the first amino acid, not to artificially enrich SLiMs requiring this initial methionine (see Supplementary Figure S1).

Taking into consideration that IDRs are rich in polar residues and proline and depleted in hydrophobic residues [17], we have also incorporated a *Preserve IDRs* option for building the decoy database. When this option is enabled, we preserve the amino acid composition of IDRs when shuffling. To do so, we use IDRs annotations from UniProt and shuffle separately the regions of the protein sequence annotated as IDRs and the inter-IDRs regions. Finally, we concatenate the shuffled regions in the same order they appeared in the original protein sequence.

### 2.3 Datasets of CRM1-binding and non-binding proteins

In two of the case studies presented, the primary motif utilized was the nuclear export signal (NES). This SLiM, around 10-15 amino-acids long, plays a key role in mediating the binding of cargo proteins to the nuclear export receptor CRM1 (also known as XPO1) [18]. We refer to this motif as NES/CRM1. To carry out our analyses, we have used two experimental datasets derived from a large-scale proteomics analysis of CRM1-interacting proteins [19]. The so-called “cargo A” dataset contains the most likely CRM1-interacting proteins identified in this previous study. Conversely, the “non-binders” dataset contains proteins for which no evidence of CRM1 interaction was obtained. To map these proteins into the human proteome version used here, we first replaced the isoform accessions with the main isoform accession. Then, we manually fixed a few accessions that have become obsolete either because they have been merged (P0CW22) or demerged (P50224, P62158, Q9P0W5, Q9Y2S0) into new accessions. The resulting “cargo A”, and “non-binder” datasets contain 543 and 884 proteins, respectively.

### 2.4 PTM integration

#### 2.4.1 PhosphoSitePlus

PTMs from PhosphoSitePlus [14] were obtained from the modification site datasets provided in the *Downloads* section of the website. These *Downloads* files provide an *Ambiguous_Site* column indicating which of the reported sites are ambiguous (i.e., they correspond to mass spectrometry peptides matching multiple proteins). Ambiguous PTM sites have been removed from the datasets used in our analyses.

#### 2.4.2 dbPTM

Experimental PTM sites from dbPTM [15] were obtained from the *DOWNLOAD* section of the website. dbPTM integrates information from 41 biological PTM-related databases as well as MS/MS-identified, PTM-associated peptides manually curated from research articles through a literature survey. While dbPTM is a valuable resource, we have noticed that it contains several instances of unexpected modifications, such as phosphorylation of alanine or glutamic acid residues.

## 3 Results and discussion

It is widely assumed that using amino acid pattern matching to search for putative SLiMs in a protein dataset will result in a significant number of biologically irrelevant (false-positive) sequence hits that match the pattern by chance. To assess the magnitude of this issue, we conducted a target-decoy comparison, which is a method similar to the one commonly used in shotgun proteomics to distinguish between correct and incorrect identifications [16]. We reasoned that, as a result of evolutionary selection, biologically relevant SLiMs would be selectively enriched in a true (target) proteome, when compared to a randomly-generated (decoy) proteome. The results of this comparison (see below) underscored the need for additional enrichment approaches to improve prediction of relevant motifs in pattern matching-based searches.

The new Wregex 3.0 incorporates several functionalities that can enhance motif prediction in the context of specific biological questions. We present three different case studies to illustrate these new functionalities.

### 3.1 Motif enrichment analysis using a decoy proteome

**Fig. 2:**
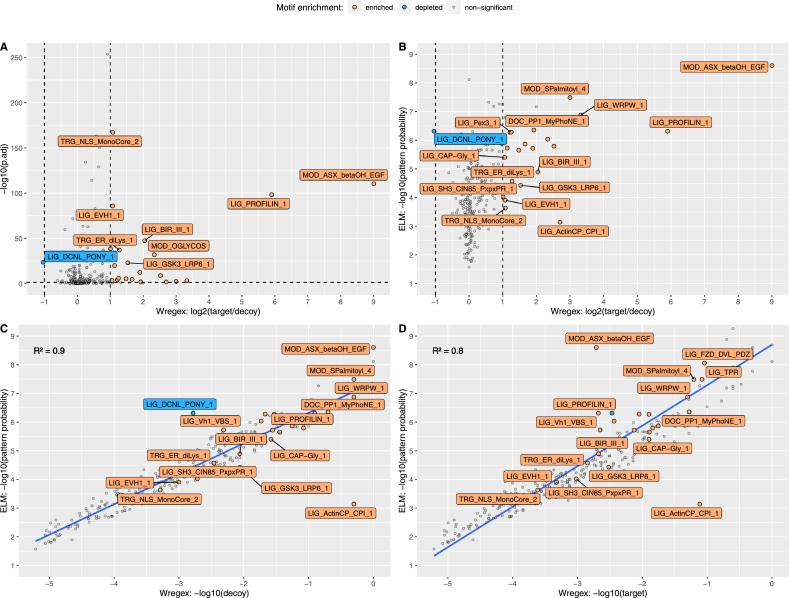
Enrichment analysis of all ELM human motifs in the human proteome and its decoy counterpart. Three cases are considered: enriched motifs when the number of matches in the target proteome is at least double that in a decoy proteome, depleted motifs when the number of matches is at most half in the target proteome compared to the decoy proteome, and non-significant cases when the proportion of matches does not meet the aforementioned criteria or when the adjusted p-value of the target and decoy proportions is above 0.05. (A) Significant motifs depicted as a volcano plot. Only 25 out of the 277 ELM motifs analyzed (9%) showed a significantly different target/decoy ratio. (B) Comparison between the ELM pattern probability and the proposed target/decoy enrichment ratio. Enriched motifs are more abundant at lower probabilities. (C and D) Correlation between the ELM pattern probability and the number of decoy and target matches respectively. The number of decoy matches represents the expected occurrences by chance according to the ELM pattern probability of the motif.

We used Wregex 3.0 to carry out pattern matching-based SLiM searches for all human ELM motifs in the entire human proteome. For each of the 277 ELM motifs that have instances in *Homo sapiens* we carried out two separated searches. The first search targeted the entire human proteome from UniProt (target), while the second search targeted a decoy “human” proteome (decoy) generated using the newly added option for decoy generation in Wregex. Remarkably, the total number of pattern matches between the target and the decoy proteomes was very similar (3022304 and 2970567 respectively). Then, for each motif we counted the number of target and decoy matches and we calculated a novel enrichment ratio defined as the ratio between the number of target and decoy matches, two sided p-values between the target and decoy proportions, and adjusted p-values using the Benjamini & Hochberg correction (FDR) for multiple comparisons. The results of this analysis are depicted in Fig. 2 and available in Supplementary Table S1.

Fig. 2A displays as a volcano plot the ratio between the number of matches in the human proteome (target matches) and its decoy counterpart (decoy matches) for every ELM human motif. A motif is considered enriched when the ratio between the number of target and decoy matches is equal or greater than 2 (1 in log2 scale), depleted when the ratio is equal or less than 0.5 (-1 in log2 scale), and non-significant when the target/decoy ratio does not meet the aforementioned criteria or when the adjusted p-value of the target and decoy proportions is above 0.05. Remarkably, only 25 out of the 277 ELM motifs analyzed (9%) showed a significantly different target/decoy ratio. This indicates that, for the vast majority of SLiMs, just matching the motif pattern does not provide enough confidence in the predicted motif. Some motifs with rare amino acid compositions such as cysteine [20] (MOD_ASX_betaOH_EGF) or those with amino acid repeats (proline repeats in LIG_PROFILIN_1) result into a high target/decoy ratio due to the shuffling of their amino acid positions in the decoy proteome.

We have repeated this analysis two more times using two new decoy databases: an additional run using an alternative strategy preserving the amino acid composition of IDRs, and a second random run using the same strategy of shuffling the full protein sequences. The results are depicted in Supplementary Figure S2 and show a similar overall target/decoy ratio distribution, thus corroborating the previous conclusions. As an exception, when preserving the IDRs composition, the LIG_PROFILIN_1 motif results into a significantly smaller target/decoy enrichment ratio (Supplementary Figure S2A), while it remains the same in the second random run shuffling the full protein sequences (Supplementary Figure S2B). This can be explained since the proline-rich regions are predominantly localized in the solvent-exposed regions such as the IDRs [21], and this composition is disrupted when shuffling the full protein sequence. Thus, shuffling the full protein sequence also highlights these special amino acid distributions in the original dataset. Finally, Supplementary Figure S2C shows that the target/decoy ratio distributes around the random case of a target/decoy ratio equal to 1 (or 0 in logarithmic scale) for the majority of the motifs, but with a larger tail on the right indicating a trend of positive selection. This distribution is very similar for the three decoy databases considered and independent of the decoy generation strategies analyzed.

In order to analyze the relation between the motif probability and the target/decoy enrichment ratio Fig. 2B uses the ELM pattern probability instead of the adjusted p-value calculated for the null hypothesis that the target and decoy proportions are the same. This ELM score reflects the probability of the regular expression to be found by chance in any given protein sequence. In this case the range of values is more convenient and it can be seen that there is a correlation between enriched motifs and the ELM pattern probability, being more enriched motifs at lower probabilities. To analyze this correlation in more depth, Fig. 2C and Fig. 2D show scatter plots between the ELM pattern probability and the numbers of decoy and target matches respectively using logarithmic scales. The correlation between the ELM pattern probability and the number of decoys is very high ($R^{2}=0.9$) because decoys represent matches just by chance, which it is also the meaning of the ELM probability. In the case of target matches, as expected, the correlation is lower ($R^{2}=0.8$) because there are non-random matches, although it is still high because there are many matches that occur just by chance. We noted that the LIG_ActinCP_CPI_1 motif does not follow the general correlation. We believe that this could be due to a miscalculation in the pattern probability provided by ELM for this particular motif. In fact, while the ELM reported value is $7.25\cdot 10^{-4}$, our manual calculation yielded a value of $7.25\cdot 10^{-8}$, which is very close to the $7.49\cdot 10^{-8}$ probability that would be obtained from the correlation chart.

These results show that the decoy strategy is a valid empirical approach for estimating random matches of motif patterns. Furthermore, the target/decoy ratio can be used as a practical measurement to quantify how different enrichment strategies enhance motif predictions in relation to random matching, as will be demonstrated in the next sections.

### 3.2 Enhancing pattern matching with PSSM-derived scores

**Fig. 3:**
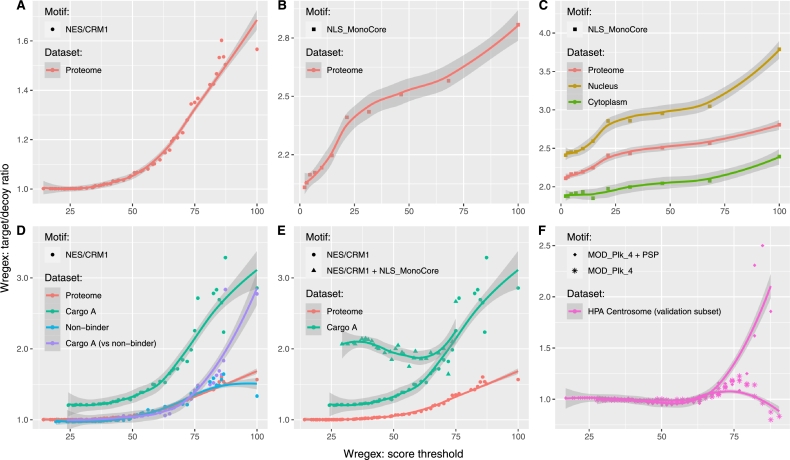
Measuring the effect of different enrichment strategies using the target/decoy ratio. (A and B) Effect of the PSSM-derived score computed by Wregex for human proteome candidates of NES/CRM1 and NLS_MonoCore motifs respectively. The use of PSSM-derived scores increases the target/decoy ratio, while the shape of the curve aids in selecting an effective score threshold. (C) Effect of subproteome enrichment for the NLS_MonoCore motif in different cellular components. Selecting a valid subproteome (nucleus) enhances the predictions in comparison to using a generic dataset such as the whole proteome. Conversely, analyzing an “incorrect” protein subset (cytoplasm) results into poorer predictions. (D) Effect of subproteome enrichment for the NES/CRM1 motif in different CRM1 cargo datasets. Cargo A motif prediction enrichment has been depicted in two configurations: using a decoy database and using a negative dataset (non-binder). In this last configuration the numbers of matches to cargo A and non-binder have been normalized to their respective database length in amino acids since they have different sizes. Selecting a valid subproteome (cargo A) enhances the predictions in relation to using a generic dataset (proteome) or an incorrect dataset (non-binder). The decoy dataset is even more negative than the non-binder dataset, which translates into a higher target/decoy ratio of cargo A case in comparison to cargo A (vs non-binder) case. (E) Enhancing NES/CRM1 motif prediction using an auxiliary NLS_MonoCore motif. The effect of the auxiliary motif is larger in the region of low scores of the main motif. (F) Enhancing MOD_PLK4 predictions with a PSSM derived from PhosphoSitePlus (PSP) phosphorylated matches in the human centrosome. The results are depicted for a validation subset different to the subset used for training the PSSM. A negative control has also been included by deriving another PSSM without considering phosphorylated matches.

We next used the target/decoy approach to evaluate to what extent the use of a PSSM-derived score, as described in the original version of Wregex [4], could enhance motif prediction. Since our main research interest is in protein nucleocytoplasmic transport, we focused on two SLiMs that have opposite roles in this process: the NES/CRM1 motif that mediates nuclear export, and the NLS_MonoCore motif that mediates nuclear import. If we just consider the sequence pattern alone, a search for NES/CRM1 motifs results in a similar number of matches in the target and decoy proteomes (target/decoy ratio = 1.01), while a search for NLS_MonoCore motif provides twice as many matches in the target (target/decoy ratio = 2.11). For these two motifs, Wregex provides a PSSM-derived score, and we tested whether applying this scoring to establish a threshold for reliable candidates may improve the prediction of these motifs. Fig. 3 shows the target/decoy ratio as a function of the Wregex score. The initial (leftmost) points on the graphs represent the efficiency of the pattern alone, without any scoring, discriminating against random matches. As shown in the graphs, using our Wregex score threshold increases the proportion of target proteins (i.e. reduces the number of motif matches just by chance). The shape of the curve suggests at which score threshold this discrimination increases. For instance, Fig. 3A shows that in the case of the NES/CRM1 motif there is not much difference using scores below 50, and the enrichment rapidly increases above this value, which is the same threshold we obtained in our original publication [4] based on experimental data. For the case of the NLS_MonoCore motif Fig. 3B shows that the score improves the prediction even from lower threshold values.

### 3.3 Subproteome enrichment

We hypothesized that an additional increase in the target/decoy ratio, reflecting a more accurate motif prediction, could be obtained by targeting a suitable (i.e. biologically relevant) protein subset for a particular SLiM. As a first example, we focused on the NLS_MonoCore motif, which is expected to be enriched in proteins located at the nucleus that need to be, in most cases, actively imported into this compartment. Using the new subproteome feature of Wregex 3.0, we conducted three distinct searches against: the complete human proteome, a human nuclear subproteome (consisting of proteins annotated with GO term GO:0005634), and a human cytoplasmic subproteome (comprising proteins annotated with GO:0005737), along with their corresponding decoys. As expected, Fig. 3C shows a higher target/decoy ratio when the presence of potential NLS motifs is analyzed in the nuclear subproteome. Conversely, the target/decoy ratio is decreased when analyzing an “incorrect” protein subset, such as the cytoplasmic proteome.

As a second example, we considered the NES/CRM1 motif, which mediates binding to the nuclear export receptor CRM1 [18]. Experimental datasets of proteins more or less likely to contain this motif are publicly available, derived from a large-scale proteomics analysis [19]. In this study, the authors identified a set of proteins (termed “cargo A”) highly likely to interact with CRM1 in an NES-dependent manner. As expected, an NES search in these “cargo A” proteins results into a higher target/decoy ratio, compared to a search in the whole human proteome (Fig. 3D). Conversely, the proteomics study also identified a set of proteins (termed “non-binders”), unlikely to bind CRM1. The target/decoy ratio for the “non-binder” dataset was very similar to the ratio obtained for the whole human proteome. We next compared the ratios between the number of matches in “cargo A” and the “non-binder” datasets. Of note, since the “cargo A” and “non-binder” datasets have a different number of proteins, the numbers of matches were normalized to the dataset size in amino acids (see Supplementary Figure S3). These analyses indicate that the decoy dataset is even “more negative” than the “non-binder” dataset, *i.e.* the number of random matches is lower, thus validating its use as a reference.

### 3.4 Enrichment using a biologically-related SLiM as auxiliary motif

We hypothesized that the prediction of a functionally relevant motif in a protein could be improved by simultaneously searching for a second, biologically-related motif. Thus, in Wregex 3.0 we have implemented the option of simultaneously searching for two motifs (which we refer to as “primary” and “auxiliary” in terms of search).

Again, we illustrate this functionality in the context of nucleocytoplasmic transport. Since proteins exported to the cytoplasm need to be initially localized in the nucleus, it seems reasonable to assume that the proportion of these exported proteins bearing a NLS should be greater in true positive matches than in false positive ones. Using the “cargo A” database as the target, we performed a Wregex search using NES/CRM1 as the primary motif and NLS_MonoCore as the auxiliary motif. As shown in Fig. 3E, NLS prediction proved useful for enhancing the predictions of candidate NESs, especially when the score of the NES motif was low.

### 3.5 Enrichment using PTM information

Finally, we tested whether the new PTM option of Wregex 3.0 can also be used for enhancing motif prediction by allowing to generate a PSSM in cases where none is available. For this analysis, we focused on the polo-like kinase PLK4 using the MOD_Plk_4 motif. Since active PLK4 is restricted to the centrosome [22], we targeted proteins located at the human centrosome. To illustrate the new Wregex 3.0 option of targeting a list of protein accessions we accessed the Human Protein Atlas (HPA) [23], and retrieved a list of 542 accession numbers of proteins that localize to the centrosome or the centriolar satellites. Next, we shuffled the accession numbers in the list and split them into two sublists with 271 entries each. These lists were used as a training set and as a test set, respectively.

We executed Wregex using only the training set and selecting *Phosphorylation* after enabling the PhosphoSitePlus (PSP) connection. We also checked the new *Filter without connections* option to remove those matches without an experimental phosphorylation in PSP. Then, we downloaded the results in fasta format (also a newly available feature) to obtain our positive training dataset file. Using the *training* tab of Wregex, we entered the following regular expression derived from MOD_Plk_4 by simply specifying the capturing groups corresponding to the desired PSSM positions [4]: (.)(.)([^IRFW])([ST])([ILMVFWY])([ILMVFWY])(.). Finally, we used the *Download Fine PSSM* button of Wregex to build the PSSM, using as input motifs the training fasta file obtained above.

To test the generalization performance of this PSSM, we used the custom motif option of Wregex to enter the regular expression and the PSSM file generated in the training step described above. Next, we performed two new searches: one targeting the test set, and another one targeting a decoy of the test set. Fig. 3F depicts the results (motif “MOD_Plk_4 + PSP”). We can see that using this PSSM enhances the motif prediction by doubling the proportion of target vs decoy matches (rightmost point) in relation to just using the motif pattern alone (leftmost point), which would result in a similar number of target and decoy matches (target/decoy ratio close to one).

Additionally, as a negative control case we repeated the steps above but now keeping those matches without an experimental phosphorylation in PSP. The results are also depicted in Fig. 3F with the motif name “MOD_Plk_4” alone and corroborate that the improvement obtained by “MOD_Plk_4 + PSP” is due to the inclusion of PTM information.

In summary, Wregex 3.0 includes a simple procedure for building a PSSM from PTM information without having a gold standard dataset. In turn, this allows to apply score thresholds that improve motif prediction reliability.

## 4 Conclusions

Here, we present Wregex 3.0, a web application for prediction of short linear motifs in proteins by enhancing the basic regular expression search with PSSM-derived scores, auxiliary motifs and experimental PTM information. Thanks to its fast execution time, Wregex can target large proteins datasets, such as the human proteome, and it provides a convenient mechanism for searching subproteomes by simply entering a GO term in the search form. Another important aspect of Wregex is its flexibility, allowing the user to use both predefined Wregex motifs, any ELM motif, and even a custom motif. The new functionalities of Wregex 3.0 have been illustrated using different case studies and evaluated using a novel methodology inspired by the target-decoy approach used in shotgun proteomics.

Wregex complements other SLiM searching tools. ScanProsite is better suited for the detection of remote similarity spreading over entire domains or proteins. SLiMSearch is a very complete tool more focused on targeting species with experimental or therapeutic relevance and providing a comprehensive list of annotations as well as calculated conservation and accessibility attributes at the expense of larger computing times. SLiMan is devoted to the analysis of interactomics results using SLiMs from ELM and SLiM-recognition domains from Pfam. Wregex is focused on searching new motif instances using a unique approach by combining a regular expression with a PSSM computed using variable length motif regions. This approach is highly computationally efficient and provides results almost instantaneously. Other unique characteristics of Wregex include the analysis of mutation impact, and the ability to build a decoy database to analyze the enrichment effect of different motif filtering approaches.

## CRediT authorship contribution statement

**Gorka Prieto:** Writing – original draft, Software, Methodology, Conceptualization. **Jose A. Rodríguez:** Writing – review & editing, Methodology, Conceptualization. **Asier Fullaondo:** Writing – review & editing, Methodology, Conceptualization.

## Declaration of Competing Interest

The authors declare that they have no conflict of interest regarding the publication of this article.

## Appendix A Supplementary material

Figure S1 N-term amino acid distribution. Amino acid counts in the first 10 positions of the 20333 reviewed proteins in the human proteome (UniProt 2023_04). Figure S1

Table S1 Enrichment results of all ELM human motifs using a decoy proteome. The target proteome is the human proteome from UniProt 2023_04 and the decoy proteome has been generated with Wregex. Results include counts of target and decoy matches, enrichment ratio, two sided p-values between the target and decoy proportions, and adjusted p-values using the Benjamini & Hochberg correction (FDR) for multiple comparisons. A motif is considered enriched when the ratio between the number of target and decoy matches is equal or greater than 2, depleted when the ratio is equal or less than 0.5, and non-significant when the target/decoy ratio does not meet the aforementioned criteria or when the adjusted p-value of the target and decoy proportions is above 0.05. Table S1

Figure S2 Effect on the target/decoy ratios of ELM human motifs when preserving the amino acid composition of IDRs in the decoy human proteome. A) Comparison of target/decoy ratios preserving IDR composition versus shuffling the full protein sequences. B) Comparison of target/decoy ratios between two random runs shuffling the full protein sequences. C) Distribution of the target/decoy ratios for the different decoy databases. Figure S2

Figure S3 Corroboration of the linear dependency assumption between the number of NES/CRM1 matches and the fasta database size in amino acids. Full range corresponds to the human proteome size in amino acids. Figure S3

File S1 A comparison of short linear motif searching tools. Similarities and differences between the Wregex 3.0, ScanProsite, SLiMSearch and SLiMan tools. File S1
