# Supplementary figures and images for: Enhancing prediction of short linear protein motifs with Wregex 3.0
Source: Comput Struct Biotechnol J. 2024 Jul 17;23:2978–84. doi: 10.1016/j.csbj.2024.07.013 (PMC11318550; doi:10.1016/j.csbj.2024.07.013)

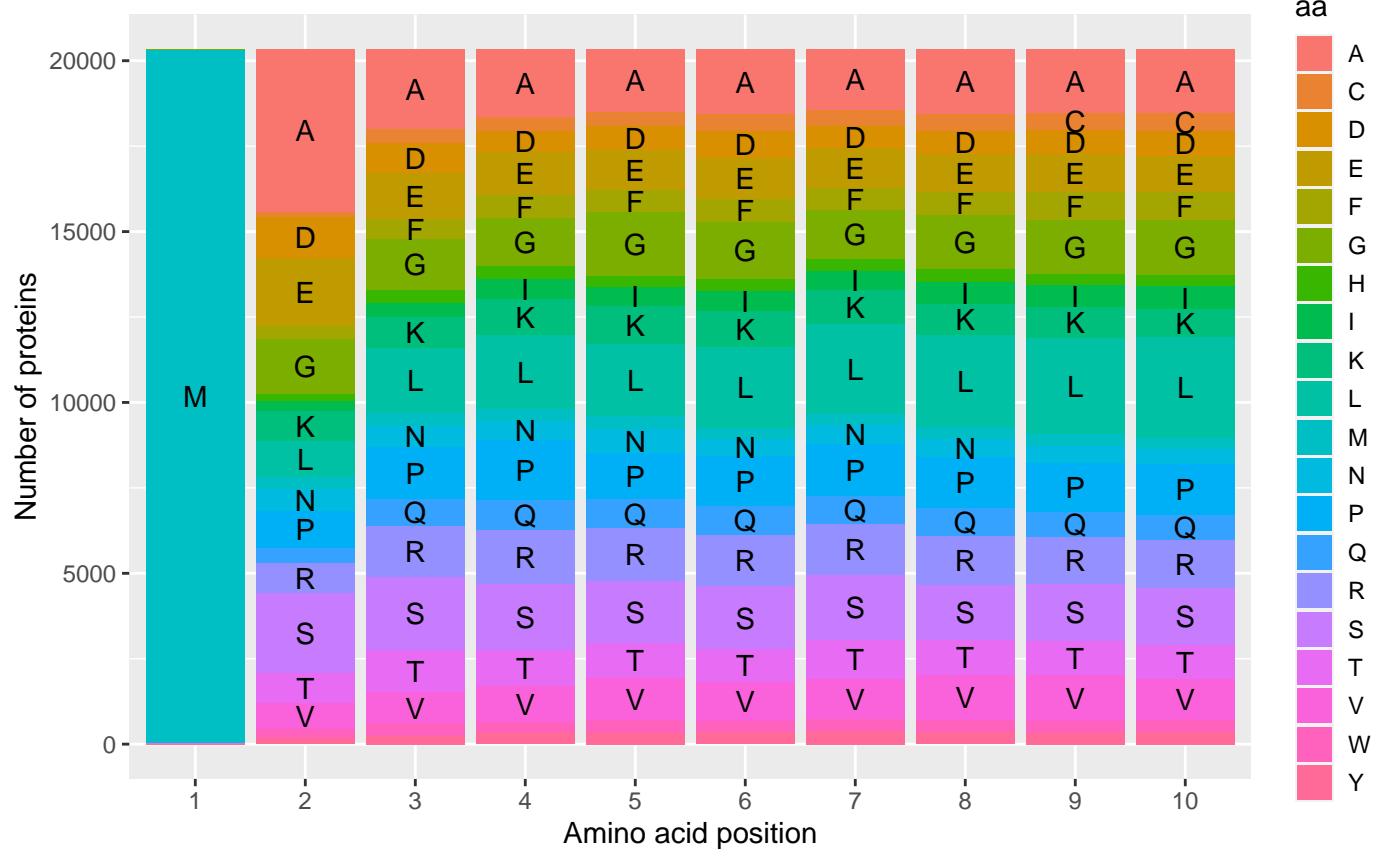

Supplement: Figure S1 — N-term amino acid distribution. Amino acid counts in the first 10 positions of the 20333 reviewed proteins in the human proteome (UniProt 2023_04). [file mmc1.pdf]

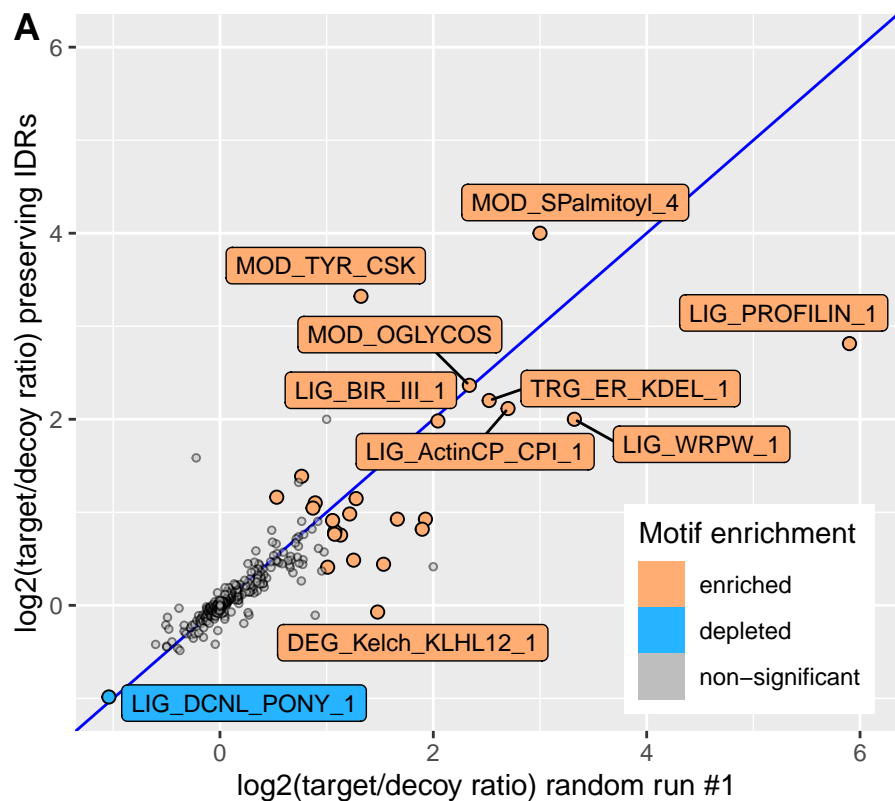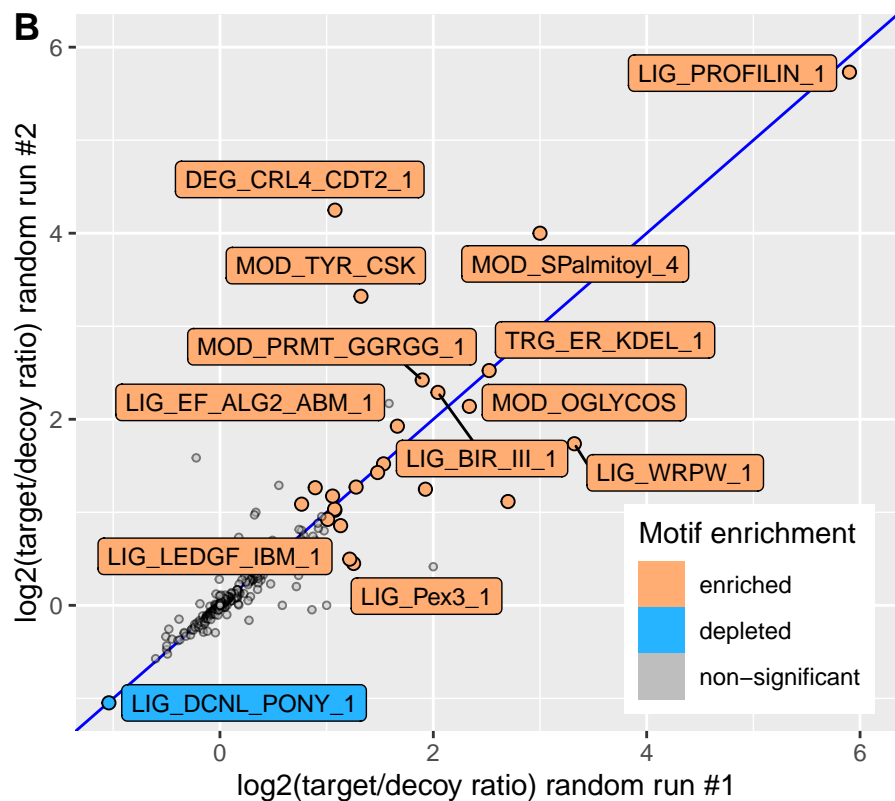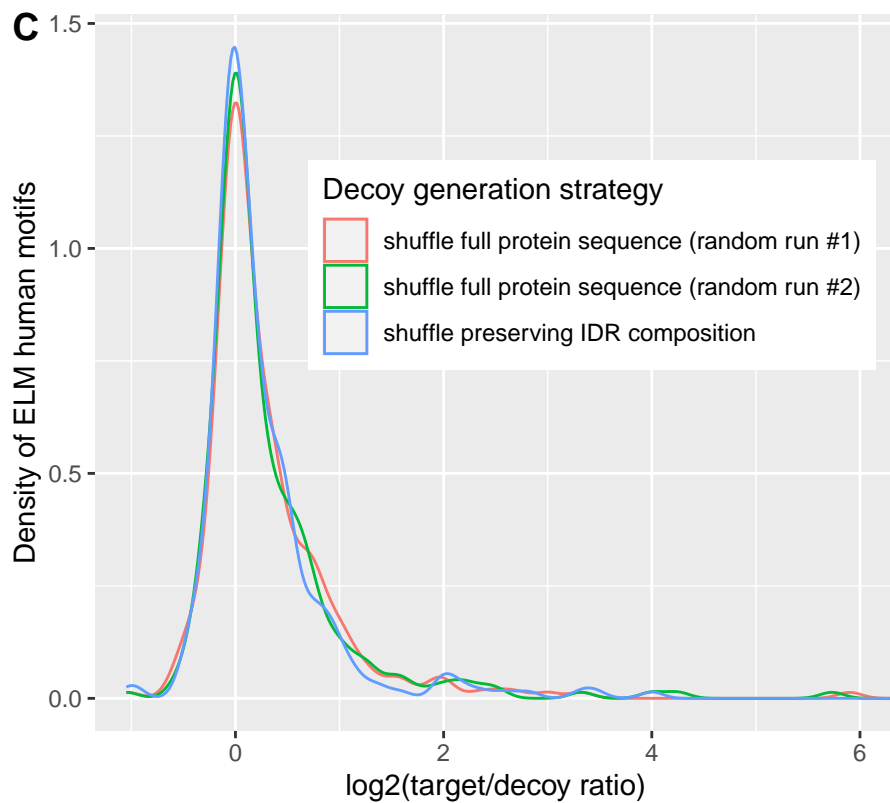

Supplement: Figure S2 — Effect on the target/decoy ratios of ELM human motifs when preserving the amino acid composition of IDRs in the decoy human proteome. A) Comparison of target/decoy ratios preserving IDR composition versus shuffling the full protein sequences. B) Comparison of target/decoy ratios between two random runs shuffling the full protein sequences. C) Distribution of the target/decoy ratios for the different decoy databases. [file mmc3.pdf]

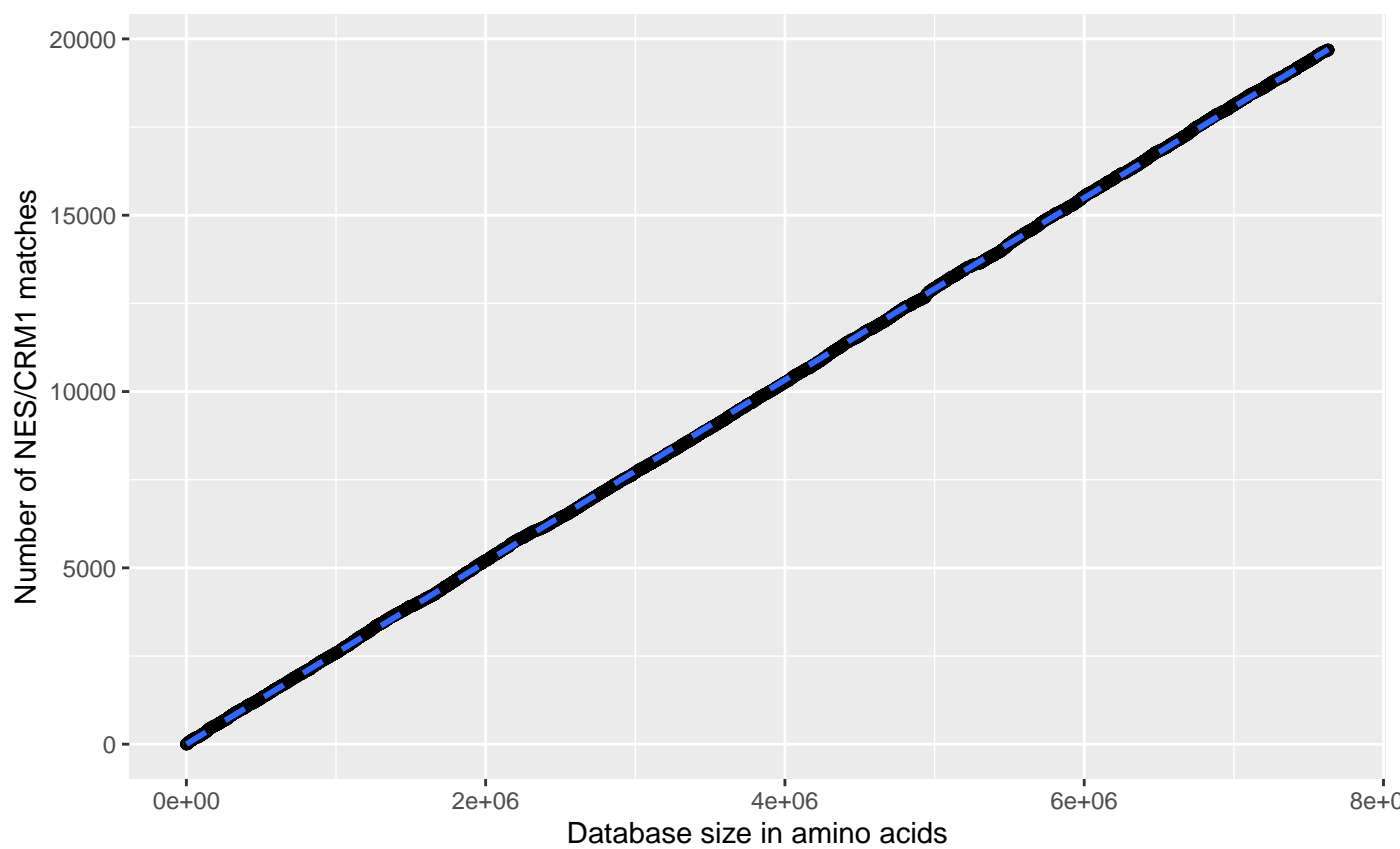

Supplement: Figure S3 — Corroboration of the linear dependency assumption between the number of NES/CRM1 matches and the fasta database size in amino acids. Full range corresponds to the human proteome size in amino acids. [file mmc4.pdf]
