# Supplementary material for: Enhancing prediction of short linear protein motifs with Wregex 3.0
Source: Comput Struct Biotechnol J. 2024 Jul 17;23:2978–84. doi: 10.1016/j.csbj.2024.07.013 (PMC11318550; doi:10.1016/j.csbj.2024.07.013)
Supplement: File S1 — A comparison of short linear motif searching tools. Similarities and differences between the Wregex 3.0, ScanProsite, SLiMSearch and SLiMan tools. [file mmc5.pdf]

A comparison of short linear motif searching tools

Gorka Prieto, Jose A. Rodríguez, Asier Fullaondo

July 10, 2024

1 Introduction

We consider the following four tools related to searching for short linear motifs (SLiMs): Wregex 3.0, ScanProsite[1], SLiMSearch[2] and SLiMAn[3]. In order to compare the inputs and outputs of the different tools, we have carried out a search using the four tools for the same question: *what SLiM instances are found for the NES/CRM1 motif in the UBP21 (USP21 gene) protein?*. We have selected this motif and protein because we are familiar with them and our goal is not to compare the performance of the tools for every motif, but to highlight the similarities and differences between the tools.

For each of the tools, we provide an explanation about its strengths and the differences with Wregex, image captures of the filled-in input search form, and the results page with a brief explanation.

2 Wregex 3.0

Wregex (Weighted regular expression) [4, 5], is a web tool that combines a regular expression with a position-specific scoring matrix (PSSM), to provide a ranked list of motif matches according to a PSSM-derived score. Wregex provides predefined PSSMs for some motifs and also allows the user to upload a training dataset to easily build a custom PSSM for any other motif. Considering that mutations that cause cancer and other human diseases may affect SLiM function, Wregex also allows predicting the impact of cancer-related missense mutations in candidate motifs, and ranking SLiMs according to the impact and prevalence of each mutation. Considering that many SLiM-mediated interactions are modulated by one or more post-translational modifications (PTMs), we have included support for enriching candidate reports with PTM information. Furthermore, considering that SLIMs are typically enriched in intrinsically disordered protein regions (IDRs) we now report whether the positions of the matched sequences fall within a disordered region. Additionally, we provide an overview of other features annotated in UniProt that intersect with the positions of the matched sequence. These features

include, among others, regions of interest, compositionally biased regions, binding sites, PTMS, secondary structures, domains and variants.

2.1 Search form

For the example under study we have selected the `NES/CRM1` motif from the *Motif* drop-down list. This motif is an extension to the original ELM regular expression including also a PSSM. In the *Target* dropdown list we have selected *List of human official gene symbols* and then entered the USP21 gene name. Finally, we have also enabled connections with COSMIC and phosphoritalation from PhosphoSitePlus. The rest of the options remains with their default values.

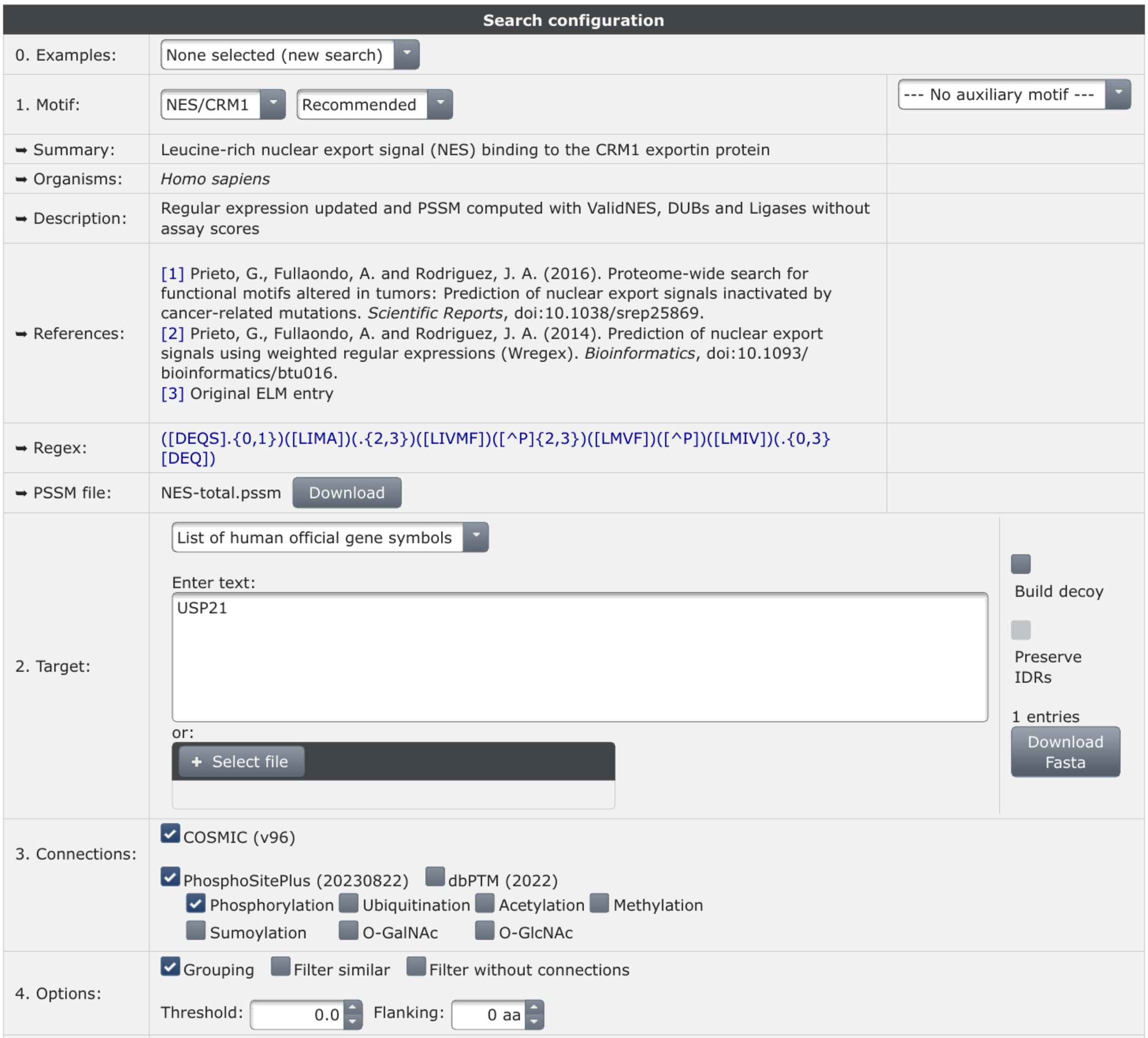

2.2 Search results

Wregex provides 8 results corresponding to different mutations in two matches: a first one with a 100.0 Wregex score in position 134..147, and a second one with a 58.8 Wregex score in position 304..316. The first one is an already described NES. Furthermore, the L144H missense mutation described is COSMIC has a maximum impact (−100.0 Wregex score difference) in the predicted NES. We already performed a full-length USP21 nuclear export assay bearing this mutation and confirmed the predicted mutation impact[5]. Wregex also reports that there is an already described serine phosphorylation within this motif at position 140.

Results: 8 results! Download CSV Download ALN Download Fasta

Search results - live scroll (scroll and wait for more results)

<table>
<tr><th>#</th><th>Entry</th><th>Motif</th><th>Start</th><th>End</th><th>Sequence</th><th>Mutant</th><th>"!"</th><th>Score</th><th>Features</th><th>Gene</th><th>COSMIC missense</th><th>Mutation impact</th><th>Phosphor...</th><th>Total PTMs</th></tr>
<tr><td>1</td><td>sp|Q9UK80|UBP21_HUMAN</td><td>NES/CRM1</td><td>134</td><td>147</td><td>E-L-GAA-L-SR-L-A-L-RPE</td><td>lost! -> elgaalsrlaHrpe</td><td>1</td><td>100,0</td><td>2</td><td>USP21</td><td>1</td><td>-100,0</td><td>1</td><td>1</td></tr>
<tr><td>2</td><td>sp|Q9UK80|UBP21_HUMAN</td><td>NES/CRM1</td><td>134</td><td>147</td><td>E-L-GAA-L-SR-L-A-L-RPE</td><td>e-l-gaa-l-sr-F-a-l-rpe</td><td>1</td><td>100,0</td><td>2</td><td>USP21</td><td>1</td><td>-15,2</td><td>1 S@140</td><td>1</td></tr>
<tr><td>3</td><td>sp|Q9UK80|UBP21_HUMAN</td><td>NES/CRM1</td><td>134</td><td>147</td><td>E-L-GAA-L-SR-L-A-L-RPE</td><td>e-l-gaa-l-sr-l-a-l-Wpe</td><td>1</td><td>100,0</td><td>2</td><td>USP21</td><td>1</td><td>-15,2</td><td>1</td><td>1</td></tr>
<tr><td>4</td><td>sp|Q9UK80|UBP21_HUMAN</td><td>NES/CRM1</td><td>304</td><td>316</td><td>EF-L-KLL-M-ER-L-H-L-E</td><td>ef-l-kll-m-er-l-h-l-D</td><td>3</td><td>58,8</td><td>3</td><td>USP21</td><td>3</td><td>+0,0</td><td></td><td>0</td></tr>
<tr><td>5</td><td>sp|Q9UK80|UBP21_HUMAN</td><td>NES/CRM1</td><td>134</td><td>147</td><td>E-L-GAA-L-SR-L-A-L-RPE</td><td>e-l-gPa-l-sr-l-a-l-rpe</td><td>1</td><td>100,0</td><td>2</td><td>USP21</td><td>1</td><td>+0,0</td><td>1</td><td>1</td></tr>
<tr><td>6</td><td>sp|Q9UK80|UBP21_HUMAN</td><td>NES/CRM1</td><td>134</td><td>147</td><td>E-L-GAA-L-SR-L-A-L-RPE</td><td>e-l-gaa-l-sr-l-T-l-rpe</td><td>1</td><td>100,0</td><td>2</td><td>USP21</td><td>1</td><td>+0,0</td><td>1</td><td>1</td></tr>
<tr><td>7</td><td>sp|Q9UK80|UBP21_HUMAN</td><td>NES/CRM1</td><td>134</td><td>147</td><td>E-L-GAA-L-SR-L-A-L-RPE</td><td>e-l-gaa-l-sr-l-a-l-Qpe</td><td>1</td><td>100,0</td><td>2</td><td>USP21</td><td>1</td><td>+0,0</td><td>1</td><td>1</td></tr>
<tr><td>8</td><td>sp|Q9UK80|UBP21_HUMAN</td><td>NES/CRM1</td><td>304</td><td>316</td><td>EF-L-KLL-M-ER-L-H-L-E</td><td>ef-l-kll-m-eL-l-h-l-e</td><td>3</td><td>58,8</td><td>3</td><td>USP21</td><td>1</td><td>+0,0</td><td></td><td>0</td></tr>
</table>

3 ScanProsite

ScanProsite detects PROSITE[6] signature matches in protein sequences and complement them with extra meaningful information from ProRule[7]. PROSITE consists of entries describing protein domains, families and functional sites, as well as associated signatures to identify them. These signatures can be either profiles (weight matrices) that describe protein families and modular protein domains, or patterns (regular expressions) that describe short sequence motifs. Each type of signature has its own purpose, but they are not combined for the same SLiM searching purpose like we do in Wregex. PROSITE profiles are better suited for the detection of remote similarity spreading over entire domains or proteins, while patterns, as used in Wregex, are more suited for the identification of meaningful amino acid residues with a functional or structural role[7].

3.1 Search form

Since there is not a pattern already defined in PROSITE for the NES/CRM1 motif, we have manually entered a custom motif based in the one we defined in Wregex[4]. ScanProsite uses it own pattern format, which may be more convenient for novice users but it is more limited than the pattern format used by the other tools, which allows more complex definitions. The Wregex NES/CRM1 pattern in PROSITE format is:

```
[DEQS]-x(0,1)-[LIMA]-x(2,3)-[LIVMF]-{P}(2,3)-[LMVF]-{P}-[LMIV]-x(0,3)-[DEQ]
```

And as a target protein we have entered the USP21 UniProt accession: Q9UK80.

○ Option 1 - Submit PROTEIN sequences to scan them against the PROSITE collection of motifs.

○ Option 2 - Submit MOTIFS to scan them against a PROTEIN sequence database.

◉ **Option 3 - Submit PROTEIN sequences and MOTIFS to scan them against each other.**

Reset

STEP 1 - Submit PROTEIN sequences [help (/scanprosite/scanprosite_doc.html#mo_prot_seq)]

◉ Submit PROTEIN sequences (max. 1'000) Examples

○ Submit a PROTEIN database (max. 16MB) for repeated scans (The data will be stored on our server for 1 month).

```
Q9UK80
```

Supported input:

- UniProtKB (https://www.uniprot.org/) accessions e.g. P98073 or identifiers e.g. ENTK_HUMAN *
- PDB (http://www.rcsb.org/pdb/home/home.do) identifiers e.g. 4DGJ
- Sequences in FASTA format (http://en.wikipedia.org/wiki/FASTA_format)

**All UniProtKB/Swiss-Prot accessions/identifiers and all UniProtKB/TrEMBL accessions/identifiers of entries belonging to reference proteomes are* [illegible]

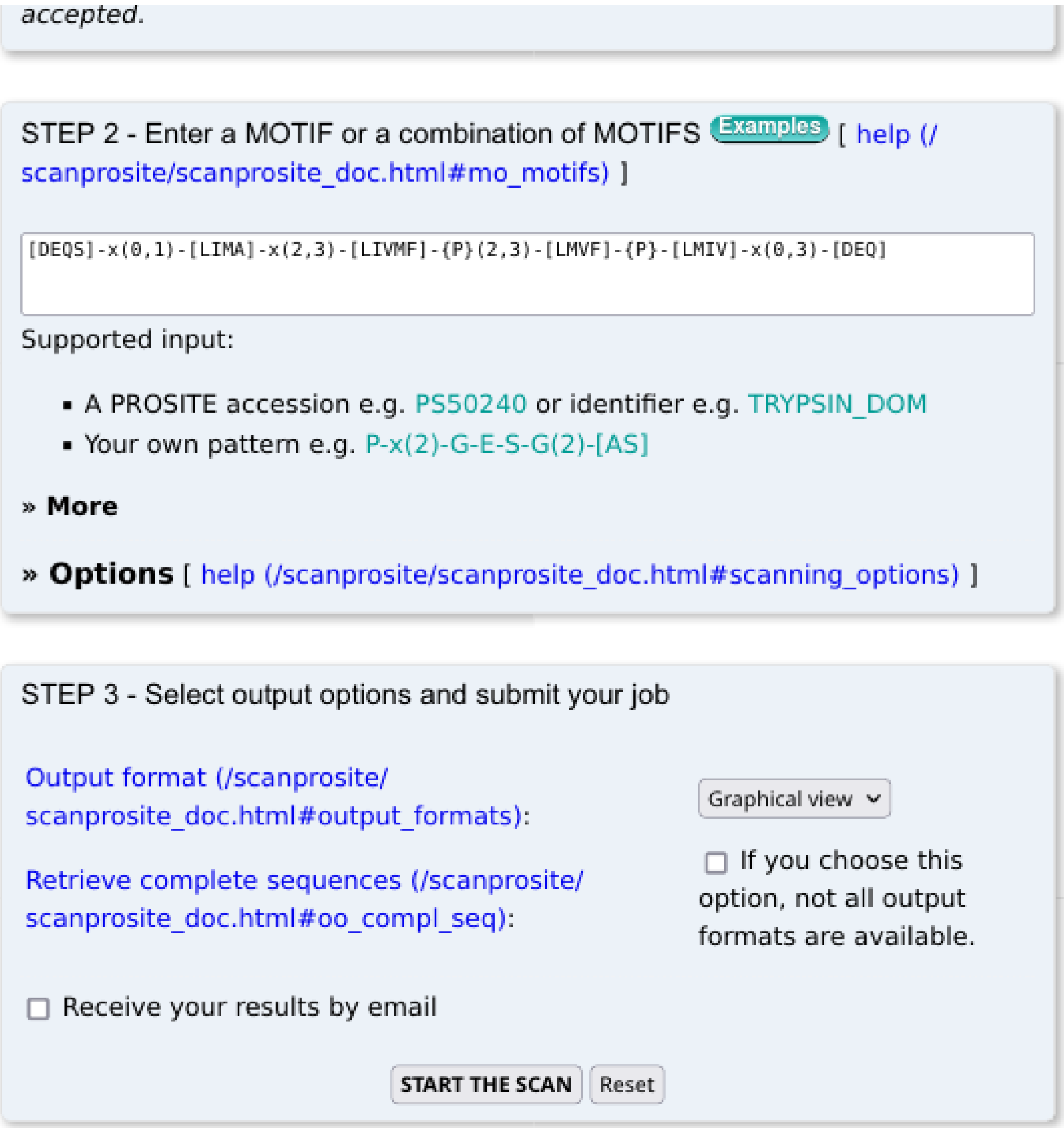

3.2 Search results

ScanProsite detects the same matches than Wregex because of the same pattern used, but it does not provide scores. While ScanProsite reports separately two overlapping matches, Wregex groups them by default (it can be disabled).

ScanProsite also reports hits for all PROSITE motifs in the full protein sequence (not within the matches specifically), resulting into one hit for the USP_3 domain profile in position 212..558, and two hits by two different patterns: USP_1 domain signature in position 213..228 and USP_2 domain signature in position 502..519.

Hits for USERPAT1 "[DEQS]-x(0,1)-[LIMA]-x(2,3)-[LIVMF]-{P}(2,3)-[LMVF]-{P}-[LMIV]-x(0,3)-[DEQ]" on sequence Q9UK80 [UniProtKB/Swiss-Prot]:

UniProtKB/Swiss-Prot (Release 2024_03 of 29-May-24) contains 571'609 entries.

found: 3 hits in 1 sequence

Q9UK80 (https://www.uniprot.org/uniprotkb/Q9UK80) **UBP21_HUMAN** (565 aa)
RecName: Full=Ubiquitin carboxyl-terminal hydrolase 21 {ECO:0000305}; EC=3.4.19.12 {ECO:0000269|PubMed:10799498, ECO:0000269|PubMed:32011234}; AltName: Full=Deubiquitinating enzyme 21 {ECO:0000303|PubMed:10799498}; AltName: Full=Ubiquitin thioesterase 21 {ECO:0000303|PubMed:10799498}; AltName: Full=Ubiquitin-specific-processing protease 21 {ECO:0000303|PubMed:10799498};. *Homo sapiens (Human)*

```
MPQASEHRLGRTREPPVNIQPRVGSKLPFAPRARSKERRNPASGPNPMLRPLPPRPGLPDERLKKL
ELGRGRTSGPRPRGPLRADHGVPLPGSPPPTVALPLPSRTNLARSKSVSSGDLRPMGIALGGHRGT
GELGAALSRLALRPEPPTLRRSTSLRRLGGFPGPPTLFSIRTEPPASHGSFHMISARSSEPFYSDD
KMAHHTLLLGSGHVGLRNLGNTCFLNAVLQCLSSTRPLRDFCLRRDFRQEVPGGGRAQELTEAFAD
VIGALWHPDSCEAVNPTRFRAVFQKYVPSFSGYSQQDAQEFLKLLMERLHLEINRRGRRAPPILAN
GPVPSPPRRGGALLEEPELSDDDRANLMWKRYLEREDSKIVDLFVGQLKSCLKCQACGYRSTTFEV
FCDLSLPIPKKGFAGGKVSLRDCFNLFTKEEELESENAPVCDRCRQKTRSTKKLTVQRFPRILVLH
LNRFSASRGSIKKSSVGVDFPLQRLSLGDFASDKAGSPVYQLYALCNHSGSVHYGHYTALCRCQTG
WHVYNDSRVSPVSENQVASSEGYVLFYQLMQEPPRCL
```

Legend:

disulfide bridge   active site   other 'ranges'   other sites

Please note that the graphical representations of domains displayed hereafter are for illustrative purposes only, and that their colors and shapes are not intended to indicate homology or shared function.
For more information about how these graphical representations are constructed, go to https://prosite.expasy.org/mydomains/ (https://prosite.expasy.org/mydomains/).

hits by patterns: [3 hits (by 1 pattern) on 1 sequence]

ruler:

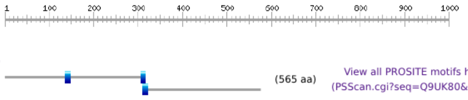

Q9UK80 (https://www.uniprot.org/uniprotkb/Q9UK80) (UBP21_HUMAN)   (565 aa)   View all PROSITE motifs hits on sequence (PSScan.cgi?seq=Q9UK80&output=nice)

RecName: Full=Ubiquitin carboxyl-terminal hydrolase 21 {ECO:0000305}; EC=3.4.19.12 {ECO:0000269|PubMed:10799498, ECO:0000269|PubMed:32011234}; AltName: Full=Deubiquitinating enzyme 21 {ECO:0000303|PubMed:10799498}; AltName: Full=Ubiquitin thioesterase 21 {ECO:0000303|PubMed:10799498}; AltName: Full=Ubiquitin-specific-processing protease 21 {ECO:0000303|PubMed:10799498};. *Homo sapiens (Human)*

USERPAT1 :
Pattern: **[D E Q S] - x (0 , 1) - [L I M A] - x (2 , 3) - [L I V M F] - { P } (2 , 3) - [L M V F] - { P } - [L M I V] - x (0 , 3) - [D E Q]**
Can't estimate number of random matches : ?

134 - 147: E.LgaaLSR.LALrp.E

300 - 311: QdAqe.FLK.LLM...E

304 - 316: EfLkllMER.LHL...E

hits by profiles: [1 hit (by 1 profile) on 1 sequence]

Upper case represents match positions, lower case insert positions, and the '-' symbol represents deletions relative to the matching profile.

ruler:

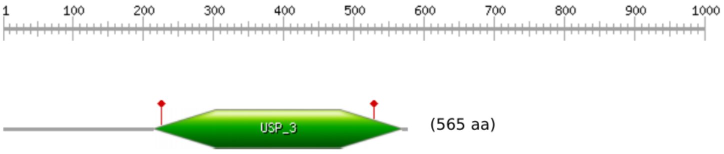

Q9UK80 (https://www.uniprot.org/uniprotkb/Q9UK80) (UBP21_HUMAN)   (565 aa)

hits by patterns: [2 hits (by 2 distinct patterns) on 1 sequence]

ruler:

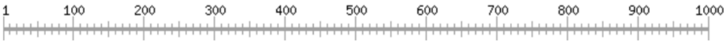

PS00972 (/cgi-bin/prosite/nicedoc.pl?PS00972) **USP_1** *Ubiquitin specific protease (USP) domain signature 1* : Hits of PS00972 on PDB 3D structures x-referenced by Q9UK80: 2Y5B_A (/cgi-bin/pdb/pdb_structure_viewer.cgi?pdb=2Y5B_A&ps=PS00972), 2Y5B_E (/cgi-bin/pdb/pdb_structure_viewer.cgi?pdb=2Y5B_E&ps=PS00972), 3I3T_A (/cgi-bin/pdb/pdb_structure_viewer.cgi?pdb=3I3T_A&ps=PS00972), 3I3T_C (/cgi-bin/pdb/pdb_structure_viewer.cgi?pdb=3I3T_C&ps=PS00972), 3I3T_E (/cgi-bin/pdb/pdb_structure_viewer.cgi?pdb=3I3T_E&ps=PS00972), 3I3T_G (/cgi-bin/pdb/pdb_structure_viewer.cgi?pdb=3I3T_G&ps=PS00972), 3MTN_A (/cgi-bin/pdb/pdb_structure_viewer.cgi?pdb=3MTN_A&ps=PS00972), 3MTN_C (/cgi-bin/pdb/pdb_structure_viewer.cgi?pdb=3MTN_C&ps=PS00972)

213 - 228: [confidence level: (0) (/scanprosite/scanprosite_doc.html#of_miniprofiles)] GLrnlGNtCFLNAvLQ

Predicted feature:

ACT_SITE (https://www.uniprot.org/help/act_site) 221   [condition: none (/rule/PRU10092?ft=1#ft1)]

PS00973 (/cgi-bin/prosite/nicedoc.pl?PS00973) **USP_2** *Ubiquitin specific protease (USP) domain signature 2* : Hits of PS00973 on PDB 3D structures x-referenced by Q9UK80: 2Y5B_A (/cgi-bin/pdb/pdb_structure_viewer.cgi?pdb=2Y5B_A&ps=PS00973), 2Y5B_E (/cgi-bin/pdb/pdb_structure_viewer.cgi?pdb=2Y5B_E&ps=PS00973), 3I3T_A (/cgi-bin/pdb/pdb_structure_viewer.cgi?pdb=3I3T_A&ps=PS00973), 3I3T_C (/cgi-bin/pdb/pdb_structure_viewer.cgi?pdb=3I3T_C&ps=PS00973), 3I3T_E (/cgi-bin/pdb/pdb_structure_viewer.cgi?pdb=3I3T_E&ps=PS00973), 3I3T_G (/cgi-bin/pdb/pdb_structure_viewer.cgi?pdb=3I3T_G&ps=PS00973), 3MTN_A (/cgi-bin/pdb/pdb_structure_viewer.cgi?pdb=3MTN_A&ps=PS00973), 3MTN_C (/cgi-bin/pdb/pdb_structure_viewer.cgi?pdb=3MTN_C&ps=PS00973)

502 - 519: [confidence level: (0) (/scanprosite/scanprosite_doc.html#of_miniprofiles)] YqLyALcnHsGsvhy..GHY

Predicted feature:

ACT_SITE (https://www.uniprot.org/help/act_site) 518   [condition: none (/rule/PRU10093?ft=1#ft1)]

4 SLiMSearch

SLiMSearch [2] is a motif discovery tool that takes as input a motif consensus in regular expression syntax and a species of interest. A sortable list of matches is returned as output with overlapping sequence annotations as well as calculated discriminatory motif attributes, such as conservation and accessibility. Additionally, PSSM support is also provided by the integrated PSSMSearch[8] tool. While SLiMSearch is a powerful and very complete tool, it also has some limitations. The motif regular expression must be entered manually instead of offering the option to select it from a list of predefined motifs. The searching process requires much more time than Wregex and, while the tool conveniently can search more than 70 datasets of species with experimental or therapeutic relevance, there is no option to search using custom sequences from a fasta file. Finally, the PSSM option is only available after the search has been completed. This PSSM requires aligned peptide sequences, which limits its ability to handle variable-length regions as Wregex does with the use of capturing groups in the regular expression.

4.1 Search form

The two basic inputs required by SLiMSearch are the motif pattern and the species. There is no option to select the pattern from a predefined list, so we have copied and pasted the pattern defined by ELM for the TRG_NES_CRM1_1 motif. We have not used the pattern defined by Wregex since we want to use the predefined peptide alignments used by SLiMSearch for the TRG_NES_CRM1_1 motif for building the PSSM after the search. There is no option to limit the search to one protein, so we have searched the whole proteome to then select the USP21 protein at the expense of extra computing time. The rest of the options remain with their default values.

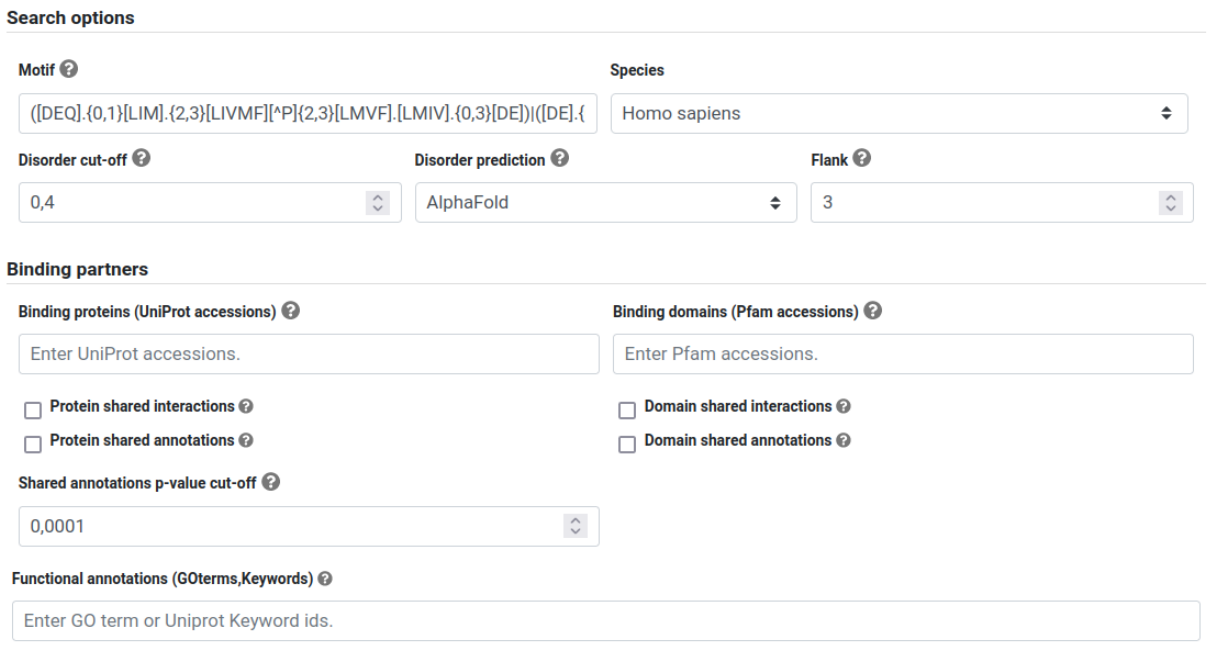

4.2 Search results

SLiMSearch also matches the 134..147 motif and provides scores related to conservation and accessibility as well as external annotations like the phosphorylation of the serine residue at position 140.

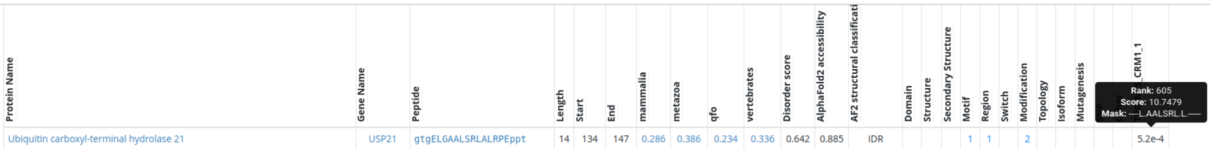

In order to include the PSSM score shown above we have navigated to the PSSM tab and selected the predefined TRG_NES_CRM1_1 motif in the examples dropdown list to use the 18 predefined aligned peptide sequences with the same length.

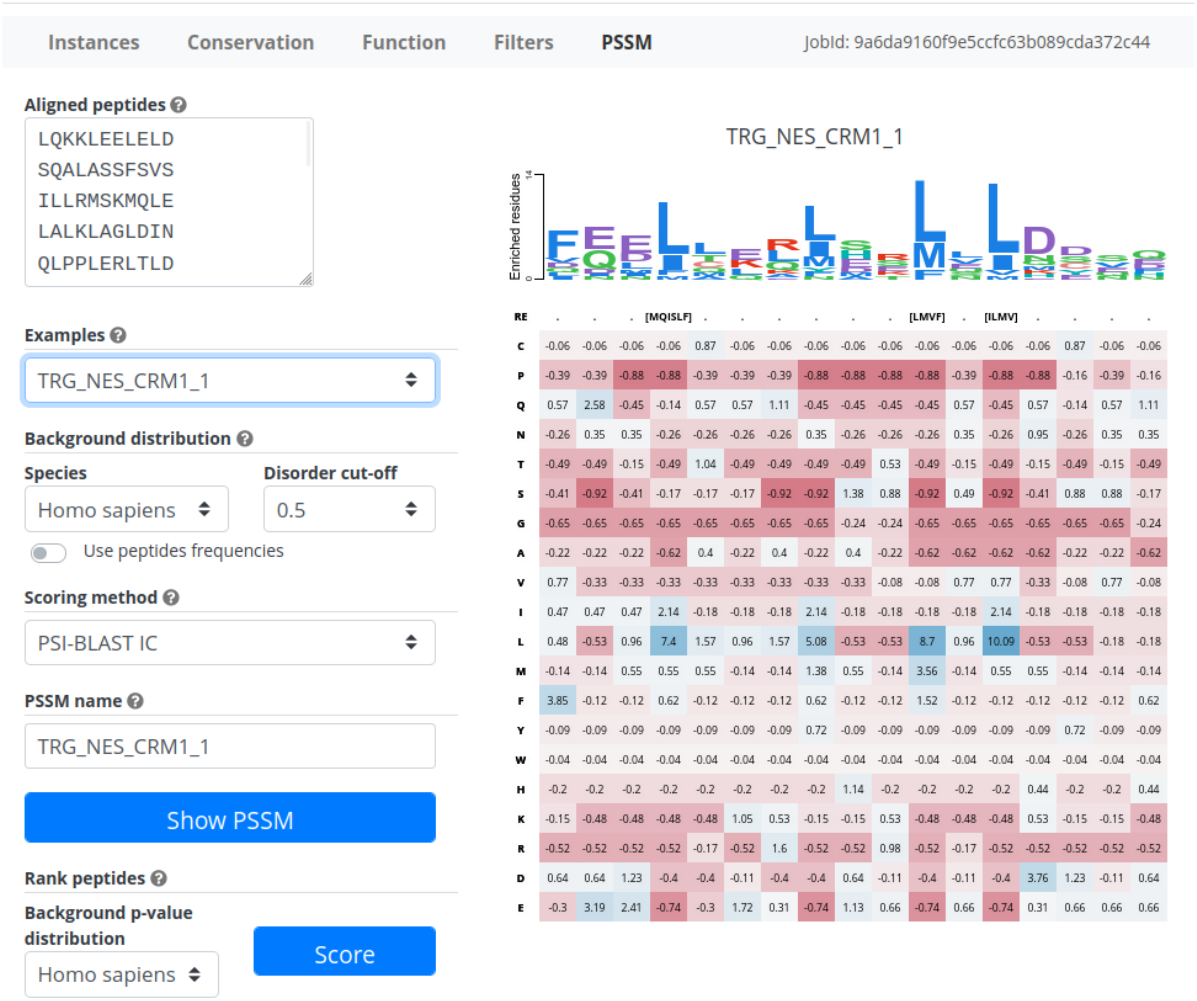

After including this PSSM the p-value of the matched motif is $5.2 \cdot 10^{-4}$, with a 10.7479 score (the best match in the proteome has a score of 19.2862).

5 SLiMAn

SLiMAn[3] is a webserver devoted to analyzing interactomics results, rather than finding new motif instances. Given a list of putative interactants, SLiMs from ELM and SLiM-recognition domains from Pfam[9] are extracted, and putative pairings are displayed. Predicted results can be filtered using motif E-values, IUPred2[10] scores, or BioGRID[11] interaction matches.

5.1 Search form

Since there is no option to enter a motif, we have entered a pair of putative interactants as UniProt accessions: our USP21 protein of interest (Q9UK80), and the human CRM1/XPO1 (O14980) which is related to our NES/CRM1 motif of interest.

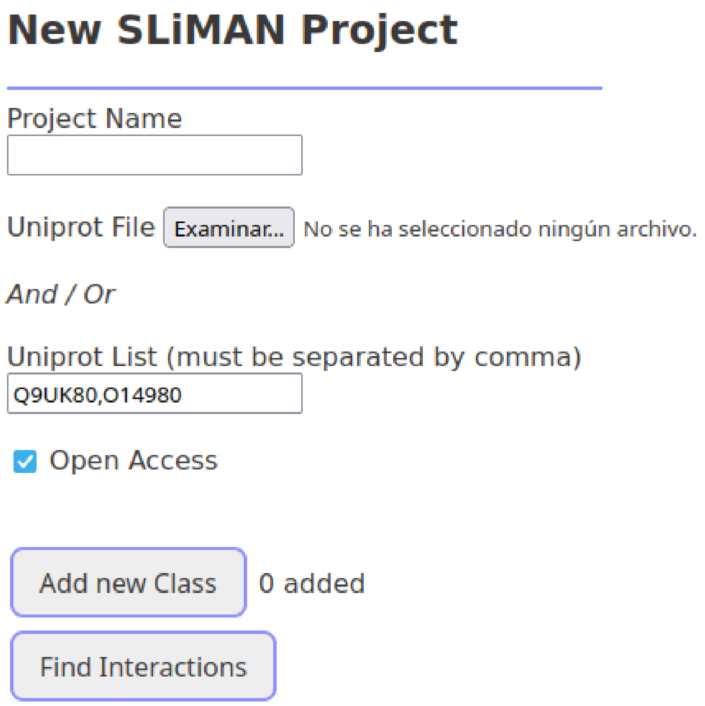

5.2 Search results

SLiMAn reports the 134..147 match corresponding to the TRG_NES_CRM1_1 ELM motif but without a match score, although it does report IUpred data. It also reports the serine phosphorylation at position 140.

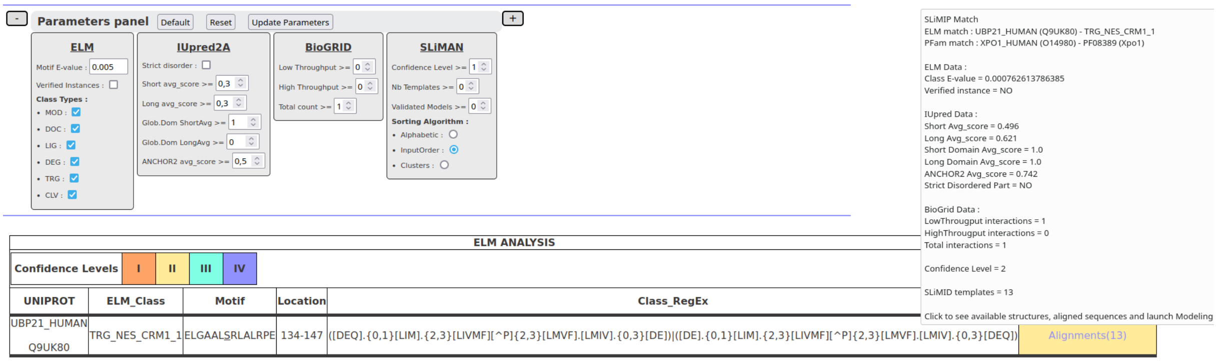

References

[1] Edouard de Castro, Christian J. A. Sigrist, Alexandre Gattiker, Virginie Bulliard, Petra S. Langendijk-Genevaux, Elisabeth Gasteiger, Amos Bairoch, and Nicolas Hulo. ScanProsite: detection of PROSITE signature matches and ProRule-associated functional and structural residues in proteins. *Nucleic Acids Research*, 34:W362–W365, 07 2006. ISSN 0305-1048.

[2] Izabella Krystkowiak and Norman E. Davey. SLiMSearch: a framework for proteome-wide discovery and annotation of functional modules in intrinsically disordered regions. *Nucleic Acids Research*, 45(W1):W464–W469, 04 2017. ISSN 0305-1048.

[3] Victor Reys and Gilles Labesse. SLiMAn: An Integrative Web Server for Exploring Short Linear Motif-Mediated Interactions in Interactomes. *Journal of Proteome Research*, 21(7):1654–1663, 2022.

[4] Gorka Prieto, Asier Fullaondo, and Jose A. Rodriguez. Prediction of nuclear export signals using weighted regular expressions (Wregex). *Bioinformatics*, 30(9):1220–1227, 01 2014. ISSN 1367-4803.

[5] Gorka Prieto, Asier Fullaondo, and Jose A Rodríguez. Proteome-wide search for functional motifs altered in tumors: Prediction of nuclear export signals inactivated by cancer-related mutations. *Scientific reports*, 6(1):1–9, 2016.

[6] Christian J. A. Sigrist, Edouard de Castro, Lorenzo Cerutti, Béatrice A. Cuche, Nicolas Hulo, Alan Bridge, Lydie Bougueleret, and Ioannis Xenarios. New and continuing developments at PROSITE. *Nucleic Acids Research*, 41(D1):D344–D347, 11 2012. ISSN 0305-1048.

[7] Christian J. A. Sigrist, Edouard De Castro, Petra S. Langendijk-Genevaux, Virginie Le Saux, Amos Bairoch, and Nicolas Hulo. ProRule: a new database containing functional and structural information on PROSITE profiles. *Bioinformatics*, 21(21): 4060–4066, 08 2005. ISSN 1367-4803.

[8] Izabella Krystkowiak, Jean Manguy, and Norman E Davey. PSSMSearch: a server for modeling, visualization, proteome-wide discovery and annotation of protein motif specificity determinants. *Nucleic Acids Research*, 46(W1):W235–W241, 06 2018. ISSN 0305-1048.

[9] Jaina Mistry, Sara Chuguransky, Lowri Williams, Matloob Qureshi, Gustavo A Salazar, Erik L L Sonnhammer, Silvio C E Tosatto, Lisanna Paladin, Shriya Raj, Lorna J Richardson, Robert D Finn, and Alex Bateman. Pfam: The protein families database in 2021. *Nucleic Acids Research*, 49(D1):D412–D419, 10 2020. ISSN 0305-1048.

[10] Bálint Mészáros, Gábor Erdős, and Zsuzsanna Dosztányi. IUPred2A: context-dependent prediction of protein disorder as a function of redox state and protein binding. *Nucleic Acids Research*, 46(W1):W329–W337, 06 2018. ISSN 0305-1048.

[11] Rose Oughtred, Jennifer Rust, Christie Chang, Bobby-Joe Breitkreutz, Chris Stark, Andrew Willems, Lorrie Boucher, Genie Leung, Nadine Kolas, Frederick Zhang, et al. The BioGRID database: A comprehensive biomedical resource of curated protein, genetic, and chemical interactions. *Protein Science*, 30(1):187–200, 2021.
